# Pharmacological risk factors associated with hospital readmission rates in a psychiatric cohort identified using prescriptome data mining

**DOI:** 10.1186/s12911-018-0653-3

**Published:** 2018-09-14

**Authors:** Khader Shameer, M. Mercedes Perez-Rodriguez, Roy Bachar, Li Li, Amy Johnson, Kipp W. Johnson, Benjamin S. Glicksberg, Milo R. Smith, Ben Readhead, Joseph Scarpa, Jebakumar Jebakaran, Patricia Kovatch, Sabina Lim, Wayne Goodman, David L. Reich, Andrew Kasarskis, Nicholas P. Tatonetti, Joel T. Dudley

**Affiliations:** 1 Institute for Next Generation Healthcare, Mount Sinai Health System, New York, NY USA; 2 Department of Genetics and Genomic Sciences, Icahn Institute for Genomics and Multiscale Biology, New York, NY USA; 3 Department of Psychiatry, Mount Sinai Health System, New York, NY USA; 4 Hackensack Meridian Health Hackensack University Medical Center, Hackensack, NJ USA; 5 Mount Sinai Data Warehouse, Mount Sinai Health System, New York, NY USA; 6 Department of Anesthesiology, Mount Sinai Health System, New York, NY USA; 7 Departments of Biomedical Informatics, Systems Biology and Medicine, Columbia University, New York, NY USA; 8 Department of Population Health Science and Policy; Icahn School of Medicine at Mount Sinai, Mount Sinai Health System, New York, NY USA

**Keywords:** Computational psychiatry, Healthcare data science, Prescriptome, Big data, Digital health, Biomedical informatics, Pharma informatics, Hospital readmission

## Abstract

**Background:**

Worldwide, over 14% of individuals hospitalized for psychiatric reasons have readmissions to hospitals within 30 days after discharge. Predicting patients at risk and leveraging accelerated interventions can reduce the rates of early readmission, a negative clinical outcome (i.e., a treatment failure) that affects the quality of life of patient. To implement individualized interventions, it is necessary to predict those individuals at highest risk for 30-day readmission. In this study, our aim was to conduct a data-driven investigation to find the pharmacological factors influencing 30-day all-cause, intra- and interdepartmental readmissions after an index psychiatric admission, using the compendium of prescription data (prescriptome) from electronic medical records (EMR).

**Methods:**

The data scientists in the project received a deidentified database from the Mount Sinai Data Warehouse, which was used to perform all analyses. Data was stored in a secured MySQL database, normalized and indexed using a unique hexadecimal identifier associated with the data for psychiatric illness visits. We used Bayesian logistic regression models to evaluate the association of prescription data with 30-day readmission risk. We constructed individual models and compiled results after adjusting for covariates, including drug exposure, age, and gender. We also performed digital comorbidity survey using EMR data combined with the estimation of shared genetic architecture using genomic annotations to disease phenotypes.

**Results:**

Using an automated, data-driven approach, we identified prescription medications, side effects (primary side effects), and drug-drug interaction-induced side effects (secondary side effects) associated with readmission risk in a cohort of 1275 patients using prescriptome analytics. In our study, we identified 28 drugs associated with risk for readmission among psychiatric patients. Based on prescription data, Pravastatin had the highest risk of readmission (OR = 13.10; 95% CI (2.82, 60.8)). We also identified enrichment of primary side effects (*n* = 4006) and secondary side effects (*n* = 36) induced by prescription drugs in the subset of readmitted patients (*n* = 89) compared to the non-readmitted subgroup (*n* = 1186). Digital comorbidity analyses and shared genetic analyses further reveals that cardiovascular disease and psychiatric conditions are comorbid and share functional gene modules (cardiomyopathy and anxiety disorder: shared genes (*n* = 37; *P* = 1.06815E-06)).

**Conclusions:**

Large scale prescriptome data is now available from EMRs and accessible for analytics that could improve healthcare outcomes. Such analyses could also drive hypothesis and data-driven research. In this study, we explored the utility of prescriptome data to identify factors driving readmission in a psychiatric cohort. Converging digital health data from EMRs and systems biology investigations reveal a subset of patient populations that have significant comorbidities with cardiovascular diseases are more likely to be readmitted. Further, the genetic architecture of psychiatric illness also suggests overlap with cardiovascular diseases. In summary, assessment of medications, side effects, and drug-drug interactions in a clinical setting as well as genomic information using a data mining approach could help to find factors that could help to lower readmission rates in patients with mental illness.

## Background

Patients with psychiatric illnesses have an increased risk for readmission to the hospital following an initial psychiatric admission, which poses several challenges for optimizing healthcare delivery [1–7]. Hospital readmission rates are evolving as a major challenge to delivering high-value and high-volume healthcare and there remains a need for innovative approaches addressing this problem. Rising readmission rates directly increase the cost, reduce the availability of clinical resources, and decrease the quality of optimized care delivery [8]. The 30-day readmission based penalization proposal by Centers for Medicare & Medicaid Services (CMS) exemplifies that healthcare providers need to use innovative and actionable methods to identify and minimize factors driving readmission to avoid penalties [9]. Several hospital quality regulatory agencies including the Agency for Healthcare Research and Quality (AHRQ) - Healthcare Cost and Utilization Project (HCUP), also considers readmission rates as a metric to evaluate the quality of care and improve patient outcome. General patient acuity risk estimators like the Charlson Comorbidity Index (CCI) [10, 11], Modified Early Warning Score (MEWS) [12], the Probability of Repeated Admission (Pra) [13], or the LACE index (a composite score of the length of stay, acuity of admission, comorbidities, and emergency department visits) are currently used to as part of the care pathways and standard of care of patient populations. While scores like the LACE have proven to be useful, these methods do not take into consideration the extensive information that could be derived from other data types, like laboratory test or prescription data. Implementation of real-time risk assessment tools coupled with automated, continuous risk estimations using heterogeneous biomedical and healthcare data could enhance the quality of health care delivery and reduce adverse patient outcomes.

### Data-driven methods to find pharmacological factors driving psychiatric readmissions

The use of computational algorithms and predictive models leveraging big data in health care could help to identify unique factors contributing to readmission in the setting of complex diseases. Applications of data-driven methods to biomedical and healthcare data has improved our understanding of new factors driving outcomes, relationship of disease comorbidities, disease subtypes, and sequelae in disease networks [14–16]. Previous studies have assessed various factors driving hospital readmission rates for psychiatric patients and found that clinical course and length of stay were associated with various socioeconomic factors including seclusion, homelessness, and community health services [17, 18]. However, these studies focused on variables based on prior clinical knowledge and a priory hypothesis, and hence lack the ability to identify novel factors driving hospital readmissions. A recent systematic review and meta-analysis of hospital readmissions has suggested that including additional parameters could improve the predictive power of models to assess readmissions [8]. Automated, predictive modeling and application of computational approaches that leverage data from electronic medical records (EMRs) and prescription records could improve the understanding of available, yet unknown factors driving complexity of patient profiles. The application of data-driven analytics and machine learning approaches has been useful for precision phenotyping, outcome prediction, treatment response prediction, and sub-type classification for various diseases [19, 20]. Various methods including machine learning based methods have already been applied to various psychiatric conditions. For example, prediction of the persistence and severity of major depressive disorder, treatment outcome prediction in depression, prediction of post-traumatic stress disorder development, and prediction of psychosis in high-risk youth [21–23]. Collectively these approaches pave a foundation for computational psychiatry that could improve the delivery of precision care to the patient populations [24–28]. In this report, we present a first attempt to evaluate the entire visit (full duration of the index admission) specific prescription data (longitudinal prescriptome) of 1275 patients hospitalized in a psychiatric unit. We also assess side effects and drug-drug interactions related to readmission within a 30-day window after the index psychiatric admission. Unlike previous analyses targeted at assessments of individual drugs or drug-class specific analytics, our approach leverages the repertoire of prescriptome data and assesses every drug reported in the cohort. Compared to traditional approaches, our method provides an unbiased view of the role of drugs in readmission risk. Furthermore, prescription data is easily available at the disposal of the care providers and can be assessed to estimate future readmission risk.

## Methods

The Mount Sinai Institutional Review Board approved the study as part of a quality control project under the theme of patient safety assessment using hospital-generated big data. An author (JJ) acted as the honest data broker to ensure privacy during the data management and analytics. The data scientists in the project received a deidentified database from the Mount Sinai Data Warehouse. All analyses were performed using the deidentified data. Data was stored in a secured MySQL database, normalized and indexed using a unique hexadecimal identifier associated with the data for psychiatric illness visits. The data pertaining to the primary encounter of admission to the psychiatric unit of Mount Sinai Hospital, NY during 2014 to indicate readmission status is encoded as a binary variable.

### Patient characteristics

The investigation cohort consists of 1275 patients, aged 18–65, and admitted for psychiatric reasons to one of the Psychiatry inpatient units of The Mount Sinai Hospital during 2014. The principal diagnosis of psychiatric illness was used to phenotype the patients in the cohort. Each patient readmitted to an inpatient unit at The Mount Sinai Hospital (psychiatric or other medical unit) within 30-days after the discharge of a psychiatry-related index admission is defined as a “case” (*n* = 89). The remainder of patients who were not readmitted to the hospital within 30-days were described as “controls” (*n* = 1186). Controls have a mean age 40.49 (50.3% male), and cases have a mean age of 38.78 (59.6% male). Collectively the cohort includes patients diagnosed with a variety of psychiatric disorders including mood disorders, suicidal ideation, psychotic disorders, etc. The most common laboratory procedures in the cohort included complete blood count, urine drug screening, gamma-glutamyltransferase, and lipase. Patients admitted to other medical facilities within the Mount Sinai Health System, other hospitals within New York city/state, other states in the country, or the rest of the world were not captured. Three authors (MMP-R, RB, and AJ) phenotyped the cohort and classified the patients into diagnostic categories as part of a quality control initiative at Mount Sinai Hospital. As an exploratory study with low case rate, no patient exclusion criteria were applied to the dataset.

### Prescriptome analyses

**Fig. 1:**
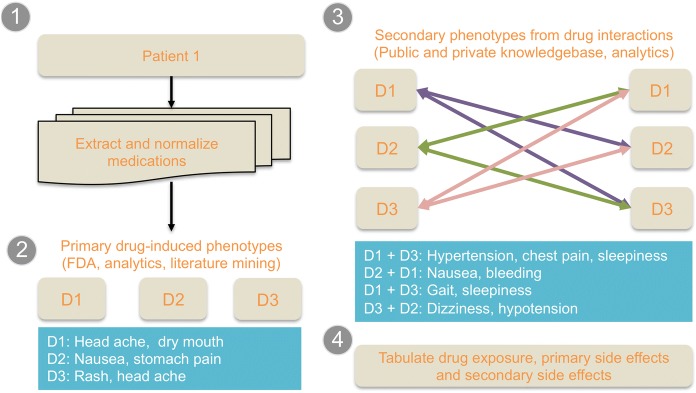
Systematic prescriptome data mining method used in the study

A flowchart of the analytical approach is provided in Fig. 1. Three modalities of pharmacological factors were assessed as follows:

1. ***Drug exposure*:** drug exposure is indicated as 1 when a drug is indicated as prescribed in the prescription record of the patient for the given visit. Data on medication adherence were not available at the time of the analyses; hence adherence level is not accounted for in the model. The drug lists were compiled and normalized using RxNorm. Individual drugs were tested using the model. Dose, mode of administration, and dose-escalation were not accounted for.
2. ***Primary side effect of individual drugs*:** Side effects of drugs were compiled from Offsides database (http://tatonettilab.org/resources/tatonetti-stm.html). Offsides [29] is a compilation of side effect data compiled from multiple databases including public databases like Food and Drug Association – Adverse Event Reporting System (FDA-AERS) https://open.fda.gov/drug/event/reference/ and SIDER [30]. A total of 1332 drugs with 10,097 side effect and 438,801 drug-effect relationships and similarities are available in the recent release of the database. Primary side effect data for individual medication was compiled from the canonical reference database and not phenotyped using EMR.
3. ***Predicted secondary side effect based on drug-drug interactions*:** Drugs often have new side effects due to interaction with other drugs [31–35]. For example, metoprolol succinate oral and ibuprofen could increase potassium levels in blood and may reduce the blood pressure level lowering effect of metoprolol. Drug-drug interactions and associated side effects can be classified as contraindications, minor, significant, and serious interactions. We compiled the drug-drug interaction across the prescriptome data using the reference database Twosides (See: http://tatonettilab.org/resources/tatonetti-stm.html). A total of 634 drugs with 1318 side effects and 4.6 million drug-drug interaction and side effect relationships are available in the recent release of the database. Secondary side effect data for drug-drug interactions were compiled from the canonical reference database and not phenotyped using EMR. For example, if a drug-drug interaction is mentioned in the reference database for any two drugs the patients were prescribed, the observation was considered as a potential side effect.

We used Bayesian logistic regression models to evaluate the association of prescription data with 30-day readmission risk. We constructed individual models and compiled results after adjusting for covariates, including drug exposure, age, and gender. All statistical analyses were performed using R language for statistical computing (http://www.R-project.org.). Data was tabulated using the *data.table* package (See: https://cran.r-project.org/web/packages/data.table/index.html) and logistic regressions were estimated using bayesglm routine in *arm* package (See: https://cran.r-project.org/web/packages/arm/index.html). Models were adjusted for multiple testing corrections using a using the Benjamini-Hochberg false discovery rate (FDR) method. Binomial proportion confidence estimates were computed across the observations and provided in the Supplemental Data for drugs, primary side effect terms and secondary side effect terms. A dedicated software package to perform pharmacological data analyses (PharmaFactors) developed for large-scale prescriptome datasets was used in this study. PharmaFactors uses an extensible analytical platform for pharmacological and prescription big data [36]. Drugs were annotated using ChemoGenomics Enrichment Analyses (CGEA) workflow [37–40]. The detailed methodology of pair-wise comorbidity estimation and shared genetic architectures is described elsewhere [14, 38].

## Results

### Patient characteristics

**Fig. 2:**
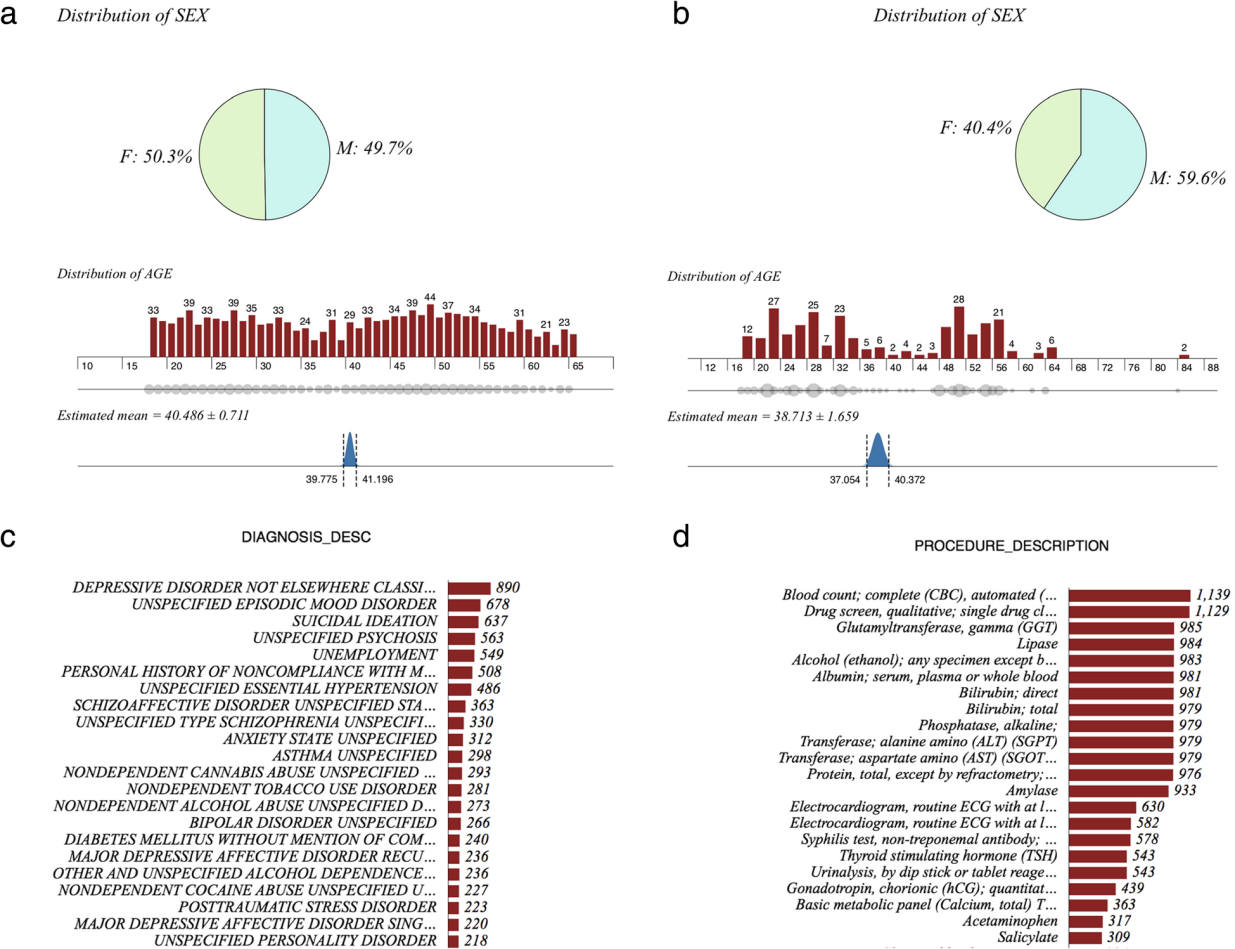
Summary of patient characteristics: **a** Gender distribution of no-readmission subset; **b** Gender distribution of readmitted subset **c** Summary of diagnoses reported from EHR **d** Summary of procedure description compiled from EHR

Patient cohort in this study includes all individuals aged 18–65, hospitalized for psychiatric complications in an inpatient psychiatric unit at Mount Sinai Hospital in New York City, NY during the year of 2014 (Fig. 2). A total of 1275 discharges were captured during this time. In the inpatient cohort, 1186 patients (no-readmission subset: 93.01%) did not have a 30-day readmission and the remaining 89 patients (readmitted subset: 6.98%) had been readmitted to the same hospital within 30 days of the index psychiatric readmission. Prescription data was compiled from EMRs. It should be noted that the lower re-admission rate is an artifact of the study design--the 10–14% national rate is rate of all readmissions per patient, whereas our rate is just re-admission to one hospital.

### Insights from prescriptome analytics

#### Drug exposure

**Fig. 3:**
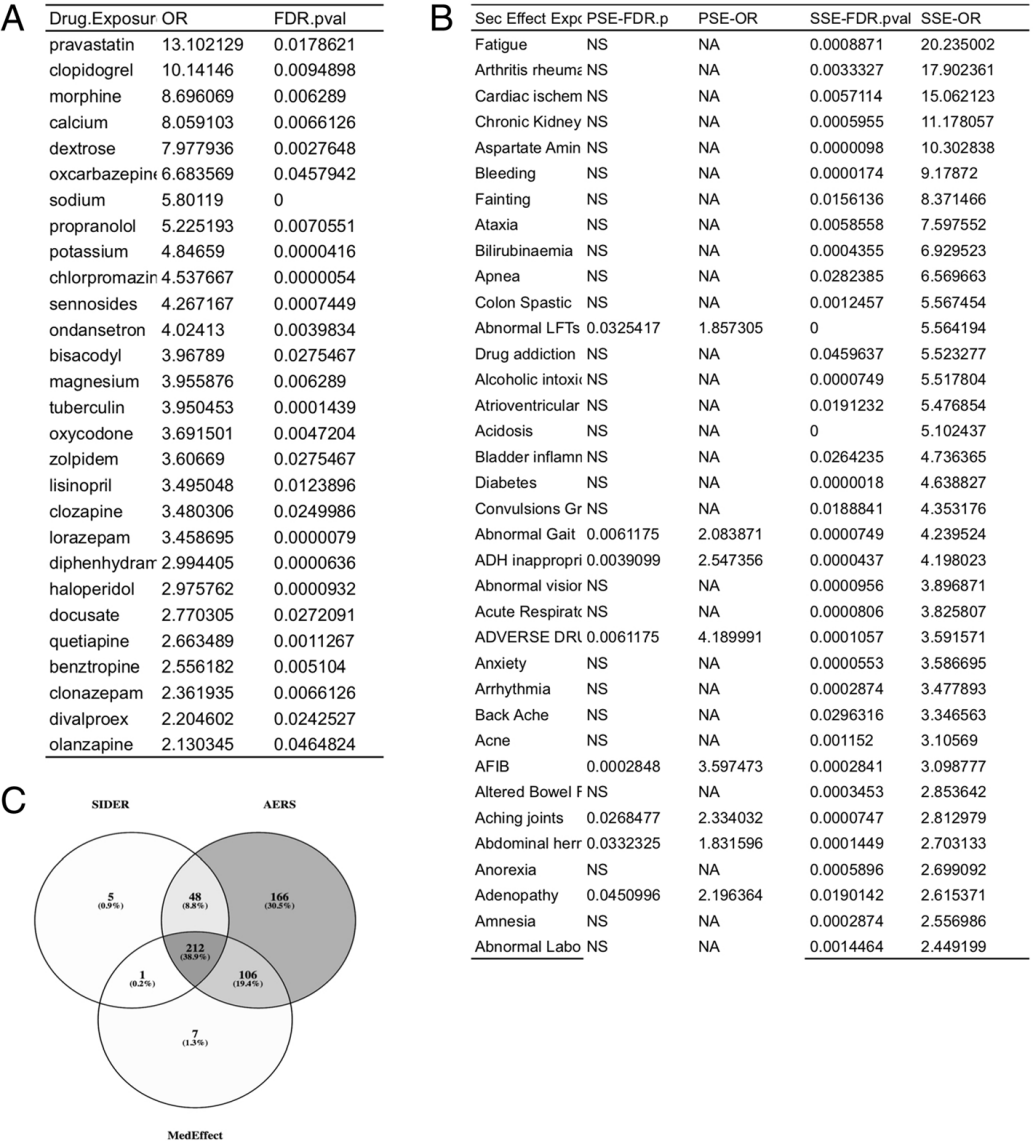
Drug exposure associated with risk for 30-day hospital readmission of psychiatric patients; full list of 888 drugs and odds ratios are provided in Supplementary Data; **a**) Individual drugs associated with readmissions **b**) Predicted secondary side effects enriched in patients readmitted to an inpatient psychiatric unit **c**) Overlap of different side effect ontologies used in the study. NS = not significant NA = the side effect term was present only in one side effect database

A total of 888 medications were prescribed for no-readmission patients and 483 medications for readmitted patients. Readmitted patients had higher mean of number of prescriptions (12.47) per patients compared to no-readmission patients (6.31) (*P* < 2.2e-16). Logistic regression models revealed that exposure to 28 drugs are significantly associated with readmission status (See Fig. 3). We have tested drug classes based on diseases and mechanism of action using Anatomical Therapeutic Chemical (ATC) classification, however these broad drug classes were not significant and hence not included as a finding.

#### Primary and secondary side effect enrichments

Side effect enrichment analyses revealed primary side effects and secondary side effects associated with readmission risk status using prescription data analytics. Exposure to pravastatin was associated with the highest odds ratio for readmission (OR = 13.10; *95% CI (2.82, 60.8); P* = 0.017;) and chlorpromazine was highly significant with moderate odds ratio (OR = 4.53; 95% CI (2.66, 7.37; *P* < 0.001). Electrocardiagram ST segment depression was one of the primary side effects associated with readmission ratio. Fatigue, rheumatoid arthritis, and cardiac ischemia were significant secondary side effects associated with readmission. A subset primary and secondary side effect are compiled in Fig. 3. The complete list of drugs, primary side effects and secondary side effects are provided in the Supplementary Data.

## Discussion

Our results provide the first line of evidence that confirms the role of current cardiovascular pharmacological treatment as an indicator of potential complexity and higher risk for early readmission in psychiatric patients [41, 42]. The impact and association of cardio-metabolic therapies and outcome of psychiatric patients has been discussed in previous studies. However, most of these studies only focused on specific drug classes (e.g. ACE inhibitors). The role of cardiovascular therapeutics to induce depression and suicidal tendencies has previously been suggested. Many commonly prescribed drugs have neurological complications as primary side effects and drug-drug interactions could lead to contraindications and further side effects due to pharmacogenomic variations [43, 44]. Also, many drugs commonly used to treat psychiatric disorders, such as antipsychotics, have known cardiovascular effects [45, 46]. Also, several antipsychotics have known cardiovascular effects [47]. Psychiatric medications are also known to cause cardiometabolic side effects including substantial weight gain, as well as adiposity-dependent and possibly adiposity-independent changes in insulin sensitivity and lipid metabolism, which increase the risk of diabetes and cardiovascular disease [20, 21]. Alternatively, cardiac medications may have direct effects on brain function. For example, the anti-thrombotic clopidogrel (OR 10.14, FDR = 0.009) disrupts neural plasticity likely by inhibiting microglial-neural interactions [48]. Individuals admitted for psychiatric reasons may be particularly susceptible to perturbation of neural plasticity by clopidogrel and alternative anti-thrombotic agents with reduced ability to cross the blood-brain-barrier, such as Ticagrelor [49], should be considered. Drug repositioning [39, 40] of the hypertension medication sodium nitroprusside has been demonstrated to have a beneficial impact on schizophrenia patients [50]. Similar beneficial effects of cardiovascular disease medication could be driven shared genetic architecture driving both diseases and warrant further targeted investigation [15, 27].

### Integrating systems pharmacology and systems biology of disease comorbidities

Drugs have pleiotropic roles in the human physiology and it is widely understood that drug-drug interactions may lead to adverse events. Balancing the efficacy and side effects are key for optimizing a treatment regime. Our systematic prescription data analytics suggests that patients prescribed with certain cardiovascular medications are at higher risk for readmission.

We noted that drug exposures significantly associated with readmission includes antipsychotics indicates multiple classes of drugs including antipsychotics (chlorpromazine, clozapine and haloperidol); ACE inhibitor (lisinopril); beta blocking agent (propranolol), antiemetics and antinauseants (ondansetron), and laxative (bisacodyl) antipruritics including antihistamines and anesthetics (diphenhydramine). From the perspective of drug-target interactions: these drugs shared multiple, common targets. For example histamine receptor H1 (*HRH1*) is a target of laxative and psycholeptics. The 5-Hydroxytryptamine Receptors (*HTR1A, HTR1B, HTR1E* and *HTR2A*) is a common target of laxatives, psycholeptics, antiemetics and antinauseants and beta-blockers. Dopamine receptors including *DRD1*, *DRD2* and *DRD3* are also targets of multiple drug classes (contact laxatives, phenothiazines with aliphatic side-chain and diazepines, oxazepines, thiazepines and oxepines). Collectively, the target space of the drugs indicates that pleiotropic drugs and drug targets may play a key role in manifesting the common side effects [38, 40]. Complete list of drugs, with their targets and mechanism of action is provided in the Supplementary Data.

**Fig. 4:**
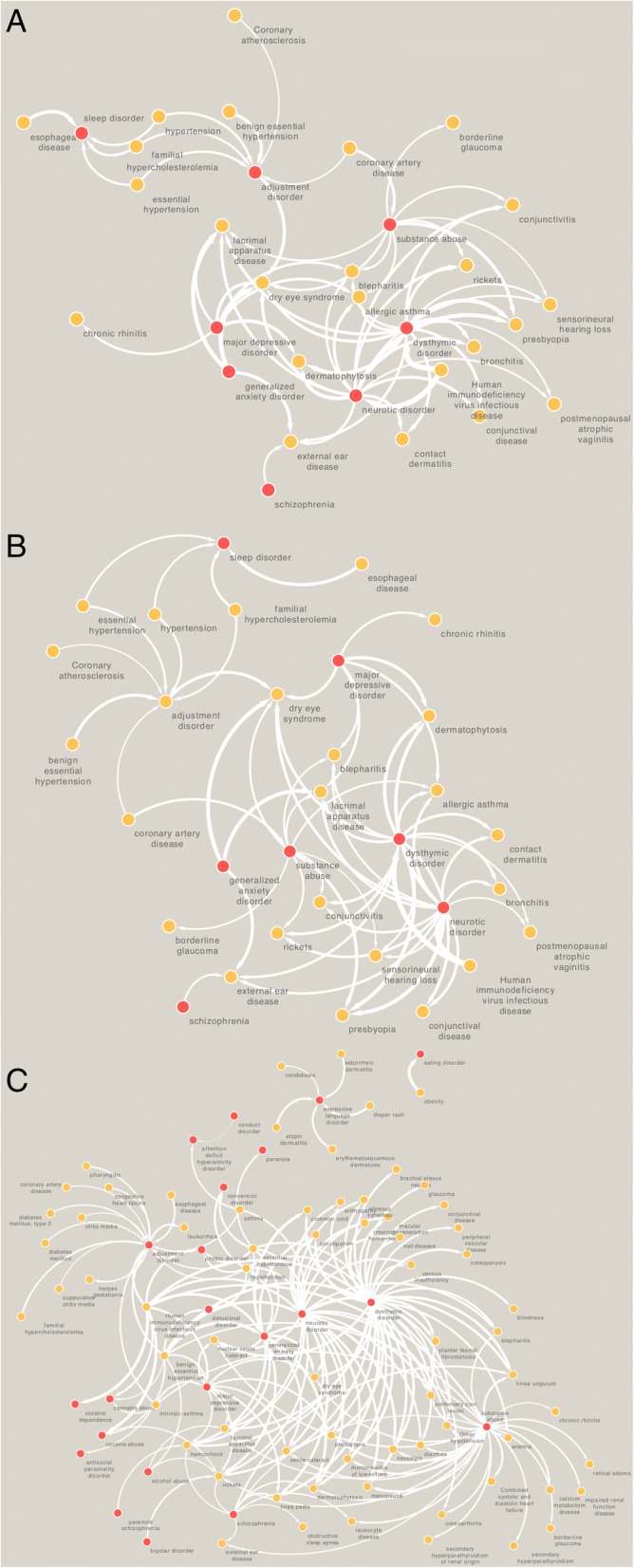
Disease network of psychiatric conditions across three different races highlighting differences in pair-wise comorbidity (**a** = European Americans; **b** = Hispanic Latinos; **c** = African Americans). Red nodes indicate neuropsychiatric disorders

To understand the epidemiological and genomic underpinnings of this finding, we have performed a digital comorbidity survey combined with assessment of shared genetic architecture between disease pairs. Based on our analyses we noticed that pair-wise comorbidity is prevalent across psychiatric and cardiovascular diseases (e.g. coronary artery disease and sleep disorder; OR = 1.75; *P =* 1.41E-09; hypertension and sleep disorder; OR = 2.82; *P =* 4.86E-46; See Fig. 4). Shared genetic architecture analyses suggest that psychiatric conditions share genetic modules with cardiometabolic diseases. For example: Schizophrenia and bradycardia (*SEMA3A, KCNJ3, KCNE1, CYP2D6, KCNH2, KCNQ1, ADRB1, KCNE2*); Schizophrenia and coronary artery diseases (*PTGS2, CRP, ACE, HP, PTGS1, AGT, ABCB1, CYP2C19, ITGB3, NOS3, MTHFR, IL6R, LTA, TNF, CYP3A4, CYP3A5, CYP2C9, IL1RN, CYP1A2, ESR1, PON1, IL6, NPY, MMP9, MMP3*); Major depressive disorder and cardiomyopathy (*CCL2, SLC6A2, ACE, SLC6A4, ADORA1, HP, EGFR, IL6, MAP 2 K1, IL1B, AGT, ADORA2A, APOE, STAT3, CYP2D6, TTR, PSEN1, PPARGC1A, ADRA2C, HTR2A, TGFB1, CTLA4, NOS3, SOD2, IFNG, CHRM2, LTA, TNF, VEGFA, AGTR2, ESR2, ESR1, IL10, GPX1, ADRB2, ADRB3, APC, AGTR1, AR*); Psychotic disorder and coronary artery disease (*TOMM40, SLC6A4, AGER, NQO1, AKT1, BDNF, ADIPOQ, DLG2, CACNA1C, ADRA2C, SOD2, ENPP1, PER2, PER1, KCNN3, CYP3A4, CYP3A5, SLC2A9, TCF7L2, NPY, MTRR, DBP, CRP, HLA-B, HLA-A, GRIA1, PDGFB, IL6, MC4R, ARNTL, ADORA2A, APOE, CYP2D6, EGR3, HTR2A, HTR2C, HRH1, SIRT1, PRKAB2, PRKAB1, PPARG, INSIG2, FGF2, FTO, IL1RN, TNFRSF1B, GSTT1, ESR1, HNF4A, PRODH, CBS, SLC22A3, VWF, ACE, CLOCK, TBX1, AKAP13, IL1B, HFE, CNR1, BSN, PDYN, MTHFR, COMT, LEPR, ADM, CYP2C9, CYP1A2, F5, GSTM1, GRIK4, CAPN10, LEP, BCL11A, PRKAA2, PRKAA1, GCLM, NPAS2, ABCB1, RGS2, NOS1, NR3C1, NOS3, MTR, TNF, PLA2G4A, PON1, GSTP1, ANK3*). The complete list of pair-wise disease comorbidities and shared genetic architecture along with drugs annotated using CGEA workflow is included in the Supplementary Data.

### Data-driven risk mitigation of hospital readmission

Implementing effective policies and strategies for reducing rates of readmission is an important quality indicator of healthcare delivery. Leveraging hospital big data for analytics and developing hyperlocal predictive models may help to predict, preempt and potentially prevent readmissions [51]. In a recent work, we have shown that machine learning-based predictive models built using EMR-wide data could augment prediction of hospital readmission [51]. Using a Naïve Bayes model build using data from diagnoses, medications, procedures and laboratory tests. Stratifying patients at-risk for cardiometabolic disease and developing a discharge process including a cardiology consultation for at risk patients may help to reduce the readmission rates. Specifically, identifying cardiovascular medications that do not cross the blood-brain-barrier may minimize side effects in this population. Stratifying patients at risk for cardiometabolic disease and developing a discharge process including a cardiology consultation for at risk patients may help to reduce the readmission rates. Furthermore, using genomic information and ascertaining pharmacogenomic and polygenic risk associated with cardiovascular disease risk and providing these to information to a psychiatry consultant may also help. Further, using genomic information and ascertaining pharmacogenomic and polygenic risk associated with cardiovascular disease risk and providing this information to psychiatry primary clinicians and consultants may also help. Further utilizing drug-drug interaction software capable of providing high-risk interactions could also help to understand and potentially reduce the pharmacological risks driven by drug repositioning [52–54].

### Clinical implications of the findings

From a clinical point of view in looking at the primary and secondary side effects with the highest OR and why clinically they may be associated with psychiatric readmission:

1. Fatigue and other quality of life related side effects might result in non-adherence with medications- resulting in increasing psychiatric symptomatology resulting in readmission.
2. Cardiovascular conditions including atrial fibrillation and cardiac ischemia- these are significant medical complications which may resulted in a medical hospitalization or at the least significant physical symptoms which increases risk of worsening psychiatric symptoms- hence resulting in increased risk of psychiatry re-admission.

## Limitations

As a proof of concept study, this study introduces large-scale prescriptome analytics method and initial results. We are in the process of replicating the finding for additional years and in another site. The overall number of patients is an apparent limitation of the current study. Furthermore, the percentage of psychiatric inpatient readmission (7.5%) observed in the cohort is lower than the national and worldwide average (10–14%). Also, the prescription data is based on prescription order, and we cannot evaluate whether patient filled it or the medication adherence. Lack of the medication adherence data is an information gap and represents an overall issue with EMR based prescription data. Given that different drugs and drug combinations contribute to identical or similar side effects, our current analytics approach is not possible to delineate individual drug-based secondary side effects due to drug-drug interactions. Performing similar analyses by integrating data from multiple years of evidence and various hospitals would further enhance the findings and allow a more robust design of interventions and policies to evaluate the role of prescription data in readmission risk. It should also be noted that the prevalence of side effects identified in the study should be accurately assessed in the target population and clinical interventions need to be adopted per hospital depending on the specific prevalence rates. In the future, predictive models to determine readmission probabilities of patients could include therapeutic features.

## Conclusions

Patients with mental illnesses have complex comorbidity profiles. Somatic comorbidities, which are common among psychiatric patients, are a potential predictor of early readmission. Inter-individual variations in acuity and comorbidity profiles exist amongst psychiatric patients. Ideally, medical care should be able to provide an optimal therapy to tailor to a specific patient’s phenotype. However, at this point our available treatments do not match the complexity of chronic diseases. We simply do not have enough tools in most cases to effectively address patients’ differences in ancestry, environmental exposures, lifestyle, etc. Thus, the delivery of precision medicine requires discovery of new predictors and algorithms to implement in a clinical setting. It is much more feasible to find and implement medications based on drug repositioning or other real-world evidence or integrate new treatment algorithms than to get a new drug certified. Understanding how medications, their side effects, and adherence patterns are related could improve outcomes in a number of different potential psychiatric cohorts. Indeed, this is an area of recurrent interest. Here, we identified drugs, primary side effects, and secondary side effects associated with readmission by mining prescription data of patients admitted to an inpatient psychiatry unit in an urban hospital. Our intriguing connection to two different cardiovascular diseases with quite large effect estimates (Odds ratios of approximately 13 and 11) suggest that cardiovascular disease is a major component that could be better managed in the psychiatric setting. It is widely understood that a number of chronic cardiovascular conditions are related to psychiatric disease. Interestingly, from our medication-based analysis it remains unclear whether the increased readmission odds are related to the medication itself and potential interactions or to the conditions, which required a physician to prescribe the medication initially. Teasing apart this relationship will be an important theme that requiring further research. In summary, we assume our analyses would direct care providers to assess the continuum of diseases associated psychiatric patients and evaluate and reconcile the medication lists, and medication adherence as a way to further reduce the readmission. This study also illustrates the impact of translational bioinformatics studies to integrate large-scale healthcare data with biological data to understand new biological insights including biological pathways, candidate genes with functional role in disease phenotypes and drug targets.

## Abbreviations

AHRQ: Agency for Healthcare Research and Quality
CCI: Charlson Comorbidity Index
CGEA: ChemoGenomic Enrichment Analyses
CMS: Centers for Medicare and Medicaid Services
EMR: Electronic Medical Records
LACE: L = length of stay of the index admission; A = acuity of the admission; C = disease comorbidities; E = number of emergency department visits in the last six months
MEWS: Modified Early Warning Score
Pra: Probability of Repeated Admission
